# Supplementary material for: Wild primates copy higher-ranked individuals in a social transmission experiment
Source: Nat Commun. 2020 Jan 23;11:459. doi: 10.1038/s41467-019-14209-8 (PMC6978360; doi:10.1038/s41467-019-14209-8)
Supplement: Supplementary file 1 — Supplementary Information [file 41467_2019_14209_MOESM1_ESM.pdf]

Supplementary Information

Wild primates copy higher-ranked individuals in a cultural transmission experiment

Canteloup et al.

Supplementary Note 1

Supplementary Table 1. Information regarding latencies of contact and success, number of observations and results of binomial tests.

First contact latency (i.e. latency to first touch one box ; in seconds and in h:m:s (cumulative latency over the course of the experiment)); first success latency (i.e. latency to first success of opening one box; in seconds and in h:m:s (cumulative latency over the course of the experiment)); number of times sli (success using lift) observed; number of individuals using sli observed; number of times spu (success using pull) observed; number of individuals using spu observed; total number of lift successes observed and total number of pull successes observed. NA indicates that the individual did not succeed in opening the box. Individuals marked with * are the ones who were opportunistically tested at the end of the experiment when the successful solvers were not around. Results of the binomial tests to test whether individuals had a preferred technique on the whole experiment are presented. P values in bold are statistically significant.

Group	Ind.	First contact latency (s)	First contact latency (h:m:s)	First success latency (s)	First success latency (h:m:s)	nb sli observed	nb inds using sli obs.	nb spu obs.	nb inds using spu obs.	Total nb lift success	Total nb pull success	P value
KB	Aare	11937	03:18:57	11958	03:19:18	12	2	17	3	2	NA	-
KB	Amur*	17747	03:15:47	48940	13:35:40	37	4	2	1	4	NA	-
KB	Arn	17064	04:44:24	39221	10:53:41	41	3	38	4	17	10	0.25
KB	Avo	396	00:06:36	582	00:09:42	0	0	3	1	235	48	**<0.0001**
KB	Lif	184	00:03:04	379	00:06:19	2	1	0	0	563	29	**<0.0001**
KB	Mal	16254	04:30:54	38699	10:44:59	58	3	17	3	15	1	**0.0005**
KB	Mis	15304	04:15:04	NA	NA	123	5	43	5	NA	NA	-
KB	Nessi	2387	00:39:47	2538	00:39:18	2	2	2	2	142	6	**<0.0001**
KB	Yalu	592	00:09:52	620	00:10:20	1	1	1	1	204	226	0.36
KB	Yan	15260	04:14:20	44468	12:21:08	50	3	25	3	5	NA	-
KB	Yeni*	49546	13:45:46	49551	13:45:51	25	4	10	3	3	NA	-
NH	Bela*	30383	08:26:23	94004	26:06:44	128	11	39	8	1	NA	-
NH	Bos	9400	02:36:40	NA	NA	123	10	41	6	NA	NA	-
NH	Can	43042	11:57:22	NA	NA	96	9	35	4	NA	NA	-
NH	Gaya	5368	01:29:28	14644	04:04:04	18	4	7	5	15	3	**0.008**
NH	Gene	129	00:02:09	132	00:02:12	0	0	0	0	494	24	**<0.0001**
NH	Gla	11180	03:06:20	11197	03:06:37	13	3	45	5	50	NA	**<0.0001**
NH	Gran	491	00:08:11	17414	04:50:14	46	5	2	1	165	84	**<0.0001**
NH	Jix	173	00:02:53	844	00:14:04	2	1	0	0	78	161	**<0.0001**
NH	Lima	22783	06:19:43	54909	15:15:09	39	7	11	4	7	3	0.34
NH	Prai	1782	00:29:42	NA	NA	73	7	12	5	NA	NA	-
NH	Pret*	42811	11:53:41	99624	27:40:24	52	9	25	4	1	NA	-
NH	Pro	23569	06:32:49	NA	NA	59	9	14	3	NA	NA	-

NH	Pru*	52480	14:34:40	90016	25:00:16	37	9	16	3	15	NA	**<0.0001**
NH	Renn*	55097	15:18:17	97005	26:56:45	22	6	2	2	9	1	**0.02**
NH	Reva	1362	00:22:42	1365	00:22:45	2	1	0	0	56	6	**<0.0001**
NH	Rey	2905	00:48:25	NA	NA	56	11	13	3	NA	NA	-
NH	Rhe	577	00:09:37	1179	00:19:39	2	1	0	0	204	45	**<0.0001**
NH	Roma	70286	19:31:26	NA	NA	2	2	0	0	NA	NA	-
NH	Rosl	23915	06:38:35	NA	NA	17	9	2	2	NA	NA	-
NH	Tir*	7784	02:09:44	90041	25:00:41	108	8	35	5	6	NA	**0.03**
NH	Twe	13742	03:49:02	NA	NA	71	6	14	3	NA	NA	-
NH	Uji	21830	06:03:50	22370	06:12:50	14	4	14	4	14	NA	**0.0001**
NH	Ula*	89954	24:59:14	97033	26:57:13	106	8	21	5	6	NA	**0.03**
NH	Umt	2802	00:46:42	9901	02:45:01	10	4	11	8	20	4	**0.002**
NH	Upps	454	00:07:34	454	00:07:34	2	1	2	2	24	1	**<0.0001**
NH	Xala	662	00:11:02	690	00:11:30	0	0	6	4	7	1	0.07
NH	Xian	4835	01:20:35	11856	03:17:36	18	5	4	2	157	25	**<0.0001**
NH	Zan	16173	04:29:33	NA	NA	28	6	19	3	NA	NA	-

Individual option preference

In a more detailed examination of the data, we note that, in Kubu, two individuals of the 12 learnt the pull option first (Table 1) but used the lift option significantly more over the course of the experiment (binomial test: $p < 0.001$; Supplementary Table 1). Eight individuals learnt the lift option first, two of them used the lift option significantly more than the pull option during the whole experiment (binomial test: $p < 0.0001$; Supplementary Table 1) while no statistically significant preferences for the two others could be detected (binomial test: Arn: $p = 0.25$; Yalu: $p = 0.36$; Supplementary Table 1); the remaining individuals displayed too few manipulation to statistically test their preference. In Noha, four individuals of the 19 first learnt the pull option (Table 1), one of them kept this preference over the course of the experiment, the other ones significantly used the lift option more than the pull option (binomial test: $p < 0.0001$; Supplementary Table 1). Fourteen individuals first learnt the lift technique, all significantly preferring to use this technique during the whole experiment (from $p \leq 0.03$ to $p \leq 0.0001$; Supplementary Table 1) except two individuals (binomial test: Lima: $p = 0.34$; Xala: $p = 0.07$; Supplementary Table 1) for which no statistically significant preferences could be detected, and two who succeeded only once to open the box using the lift option.

Supplementary Note 2: Testing for option-specific social learning

Model description

Network-based diffusion analysis (NBDA) infers social transmission of novel behaviour if the pattern of its spread follows a social network, which is taken to represent opportunities to learn from others [S3]. We used the order of acquisition (OADA) variant of NBDA [S4], which

takes as data only the order in which individuals acquire the target behaviour and not the times of acquisition. NBDA has been expanded in a number of ways, which we utilise here. First, one can include a dynamic network that changes over time [S5]. One type of dynamic network that can be used is a record of who has observed whom prior to each acquisition event. This offers the most direct measure of opportunities for learning in cases where the target behaviour is only performed at a specific location(s) that can be monitored closely [S5, S6]. In practise, a proxy of observation is used by ascertaining, for each performance of the target behaviour, who had the opportunity to observe. In this case we identified observers as individuals with their head or body oriented in an unobstructed line towards the subject manipulating the box. The use of a proxy for observation is not an inherent problem, since Hoppitt [S6] has shown that error in identifying observers does not increase the risk of a spurious social transmission effect but acts to make estimates of the strength of social learning conservative.

A second extension of NBDA is to include multiple options for solving a task [S4]. In a normal OADA, the parameter values are optimised to maximise the power of the model to predict which individual will be the next to learn the target behaviour at each acquisition event. If we extend the OADA to multiple options the parameter values are optimized to predict the combination of which individual will be next to learn and which option they will learn to use (assuming the options are including in the same stratum, see below). Thus, a multi-option NBDA differs from statistical approaches that aim to infer the presence or absence of social learning based on the option used alone [S7] - rather this information is added to the pattern of spread across the network when quantifying the strength of social transmission. It is also important to note that an NBDA extended to multiple options is not intended to model the role of social learning in the development of a preference for specific options over time, once

both are acquired to the repertoire (cf. experience weighted attraction models, S8). Rather, it is intended to model the acquisition of different behavioural variants to the behavioural repertoire, and address whether or not their acquisition to repertoire is independent (see below).

A third extension of NBDA is to include multiple networks representing different pathways of learning [S9]. This enables researchers to test whether the rate of social transmission differs between different pathways. Here we included two different pathways of learning: option-specific social learning and cross-option social learning. Option-specific (OS) social learning occurs when observation of a task solution using the lift option increases the rate at which the observer learns the lift option, and likewise for the pull option. Conversely, cross-option (CO) social learning occurs when observation of a task solution using the lift option increases the rate at which the observer learns the pull option, and vice versa. To accomplish this each individual was represented in both social networks twice- once as a learner of lift and once as a learner of pull. Henceforth we refer to 'lift individuals' and 'pull individuals', though these are the same set of individuals represented twice in the analysis, once for each option- this is simply a convenient way of coding the model expressed in Supplementary Eqn. 1 below. In the OS network, each lift individual received incoming connections based on the number of observations of lift, and each pull individual received incoming connections based on the number of observations of pull. Conversely, in the CO network, each lift individual received incoming connections based on the number of observations of pull, and vice versa for pull "individuals". In a standard NBDA the s parameter estimates the rate of social transmission per unit connection relative to asocial learning. In our model each network has an associated s parameter, denoted s_{OS} and s_{CO}. We fit models with a) a different rate for each pathway($s_{OS} \neq s_{CO}$); b) option-general social transmission ($s_{OS} = s_{CO}$);

c) OS social learning only ($s_{CO} = 0$); d) CO social learning only ($s_{OS} = 0$); and e) asocial learning ($s_{OS} = s_{CO} = 0$), allowing us to test the strength of evidence for each hypothesis (see below).

The order of acquisition (OADA) variant of NBDA [S10] takes as data only the order in which individuals acquire the target behaviour and not the times of acquisition. This has the advantage that it does not make any assumptions about the baseline rate function unlike the time of acquisition variant (TADA) [S10]. Whilst TADA can be easily modified to include increasing or decreasing baseline rates of learning, it seems likely that the rate of learning will have fluctuated with external conditions, e.g. differences in the local conditions at which the task was positioned each day. Consequently, the different groups Kubu and Noha were treated as separate diffusions in different strata in the analysis (with different unspecified baseline functions) since they were subject to different daily local conditions [S4]. However, we included diffusion of the lift and pull options in the same stratum since they are likely to have been affected in the same way by such factors. This also gives us greater power to detect OS social transmission since the model is sensitive to the order across options, e.g. if one option diffuses through a group first, followed by the other option, it adds to the evidence that individuals were learning by OS social learning. We controlled for the possibility that such a pattern might be caused by vervet monkeys learning one option more easily by asocial learning by including *option* as a factor influencing the rate of asocial learning.

We also wanted to allow for the possibility that vervet monkeys might generalize their learning, i.e. learning to solve the task using one option might increase the rate at which they subsequently learned the other option by asocial learning. Such an effect, if not controlled for, might obscure an OS social learning effect. Alternatively, learning one option might inhibit

learning of the other, which would reinforce formation of group-level traditions. To investigate this, we included a binary time-varying individual-level variable (ILV) "other-option" affecting asocial learning. For a lift individual other-option (t)= 1 if the corresponding individual had learned the pull option prior to time t, and =0 otherwise, and vice versa for pull individuals (Supplementary Table 2). If a generalization effect were operating in concert with OS social transmission, we would expect a different pattern of diffusion to option-general social transmission ($s_{OS} = s_{CO}$). e.g. If we had option-general social transmission, individuals that had observed many lifts are likely to be the next individual to solve, using either the lift or the pull option. If we have OS social transmission, paired with a generalization of learning, individuals that had observed many lifts are likely to be the next individual to solve specifically using the lift option, and once they have done so, are *then* likely to be the next to solve using the pull option. Thus different orders of events support different combinations of learning processes.

There were a number of other individual-level variables that we included as potentially having an effect on the rate of asocial and/or social learning: sex, age category (adult *versus* non-adult) and rank (quantified using the I&SI method: [S11]). We used the "unconstrained" model to include the effects of ILVs, which independently estimates the effects each ILV has on asocial and social learning [S4]. All variables were standardized so they were centred on zero, with a range of 1. Since *s* parameters are estimated relative to baseline asocial learning rate (when all ILVS=0), this transformation means they are estimated relative to an individual who is central with respect to these three ILVs. Thus, we had a total of 5 ILVs potentially having an effect on asocial learning and 3 ILVs potentially having an effect on social learning (Supplementary Table 2).

The full model can be expressed as follows:

$$\lambda_{ik1}(t) = \lambda_{0,k}(t)\left[\left(s_{OS}\sum_j o_{ij1}(t) + s_{CO}\sum_j o_{ij2}(t)\right)exp\left(\gamma_1 x_{1,i} + \gamma_2 x_{2,i} + \gamma_3 x_{3,i}\right)\right.$$
$$\left. + exp\left(\beta_1 x_{1,i} + \beta_2 x_{2,i} + \beta_3 x_{3,i} + \beta_z z_{2,i}(t)\right)\right]\left(1 - z_{1,i}(t)\right)$$

$$\lambda_{ik2}(t) = \lambda_{0,k}(t)\left[\left(s_{OS}\sum_j o_{ij2}(t) + s_{CO}\sum_j o_{ij1}(t)\right)exp\left(\gamma_1 x_{1,i} + \gamma_2 x_{2,i} + \gamma_3 x_{3,i}\right)\right.$$
$$\left. + exp\left(\beta_1 x_{1,i} + \beta_2 x_{2,i} + \beta_3 x_{3,i} + \beta_z z_{1,i}(t) + \beta_p\right)\right]\left(1 - z_{2,i}(t)\right)$$

Supplementary Equation 1.

Where $\lambda_{ikl}(t)$ is individual *i* in group *k*'s rate of acquisition of option *l* at time *t*, $\lambda_{0,k}(t)$ is the baseline rate function for group *k*, s_{OS} is a fitted parameter estimating the relative rate of OS social learning, s_{CO} estimates the relative rate of CO social learning, $o_{ijo}(t)$ is the number of observations *i* has had of *j* performing option *l* prior to time *t*, $x_{1,i}$ is the sex of *i* (0.5= female, -0.5= male), $x_{2,i}$ is the age category of *i* (0.5= adult, -0.5= non-adult), $x_{3,i}$ is the rank of *i* (ranging from -0.5 for top rank to 0.5 for bottom rank in the group), β_m are fitted parameters estimating the effect each ILV has on asocial learning, whereas γ_m estimate the effect on social learning, $z_{o,i}(t)$ is the status of individual i with respect to option *l* at time t (1= learned option; 0= naïve), β_z is a fitted parameter estimating the effect on asocial learning of having solved the other option, β_p estimates the difficulty of solving the pull option relative to the lift option.

We used a multi-model inference approach using Akaike's Information Criterion corrected for sample size (AIC_c) [S12] to obtain support for each hypothesis: a) a different rate for each pathway ($s_{OS} \neq s_{CO}$); b) option-general social transmission ($s_{OS} = s_{CO}$); c) OS social learning only ($s_{CO} = 0$); d) CO social learning only ($s_{OS} = 0$); and e) asocial learning ($s_{OS} = s_{CO} = 0$), allowing us to test the strength of evidence for each hypothesis. For each of a-d we fit models with every combination of 5 ILVs affecting asocial learning and 3 variables affecting social learning, resulting in 256 models for each set. For e) asocial learning, γ parameters have no effect so were excluded resulting in only 32 models. We calculated the total Akaike weight as a measure of support for each hypothesis a-d [S13]. Due to the lower number of models in the asocial set (e) we do not use the total Akaike weight as a measure of support for asocial learning, instead we use the 95% confidence intervals for the s parameters to this end (see below). We calculated model averaged estimates, unconditional standard errors, and the total Akaike weight for the effect of each ILV on asocial learning (β parameters) and social learning (γ parameters). In some models, standard errors could not be derived. When calculating the unconditional standard error, the standard errors for these models were replaced with an Akaike-weighted mean across models with a standard error, allowing an approximate unconditional standard error to be calculated.

Standard errors are often a misleading measure of precision for s parameters, since these often have much higher precision for a plausible lower limit than for a plausible upper limit. Consequently, we obtain 95% confidence intervals (CIs) for s parameters using the profile likelihood method. For s_{OS} we used the model with the lowest AIC_c. Since this model did not contain s_{CO}, we added the parameter to the best model in order to get 95% CI for s_{CO}, and

for $s_{OS} - s_{CO}$, quantifying the extent to which social learning is option-specific. Since s parameters are difficult to interpret directly, we also obtained an estimate of the number of learning events that are predicted to have occurred by each pathway corresponding to the estimate for each s parameter and its 95% CI [S6, S14].

Validation of method for detecting option-specific social transmission

Hoppitt, Boogert and Laland [S9] showed in a general case that inclusion of potentially confounding variables in an NBDA can statistically control for their effects and prevent spurious social transmission effects being detected. Thus, in principle, if one option is easier to learn asocially this should not result in a spurious OS social transmission effect in the NBDA, since we include a variable accounting for a potential difference in rate of asocial learning between the two options. However, to be certain that the statistical control is effective in this specific case, we ran simulations in which the observed bias towards lift was assumed to be entirely due to an asocial bias. We simulated the option choice based only on this bias. We retained the same pattern of observations but, in our simulations, these had no effect on option choice. We then fitted the same model that was favored in the real data (one in which social transmission was option specific only), and the equivalent model of option-general learning. We also fitted another two models in which an asocial bias for one or other option was added- to statistically control for its effects as we did in the real analysis. We then recorded the AICc for the best option general model minus the AICc for the best option specific model as measure of evidence for an option specific effect in the simulated dataset. We then repeated this process 1000 times, and calculated the proportion of simulations in which the evidence for option specific social transmission exceeded that observed in the real

data, and found it to be 0.0475- thus confirming that our finding for option specific social transmission is unlikely to be a result of a preference for one option.

Additional results

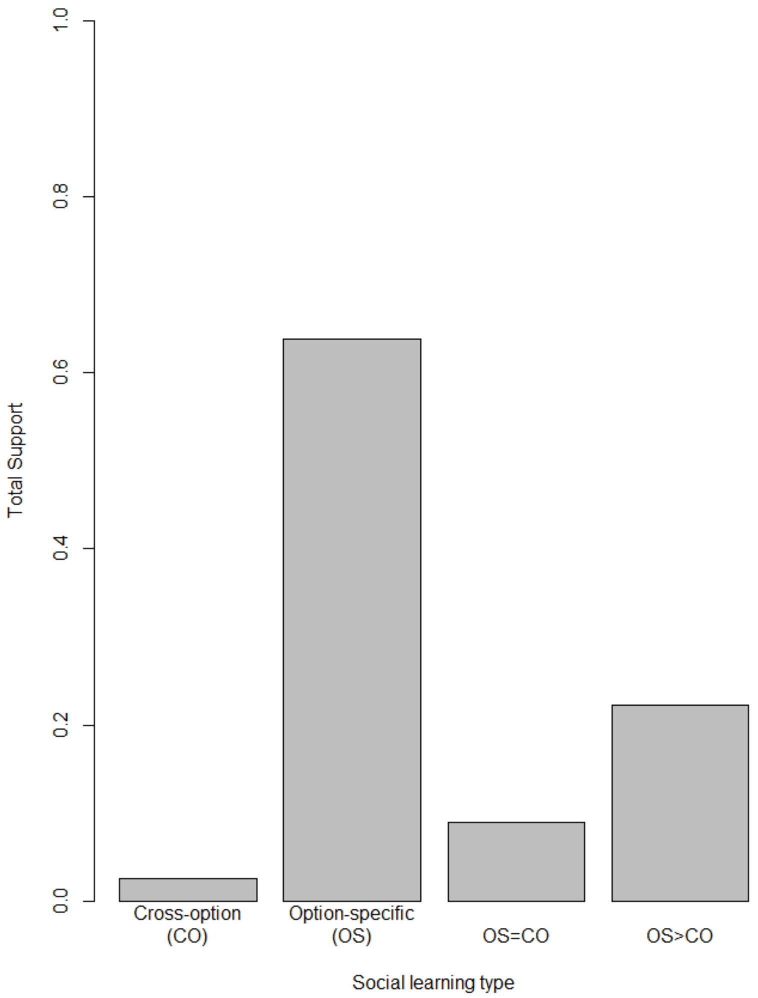

Supplementary Figure 1. Support for different combinations of option-specific (OS) and cross -option (CO) social learning. OS social learning received the most support, followed by models with separate *s* parameters for OS and CO social learning: in these cases, s_{OS} was estimated to be greater than s_{CO}. Overall these results indicate evidence that there was an OS social learning effect.

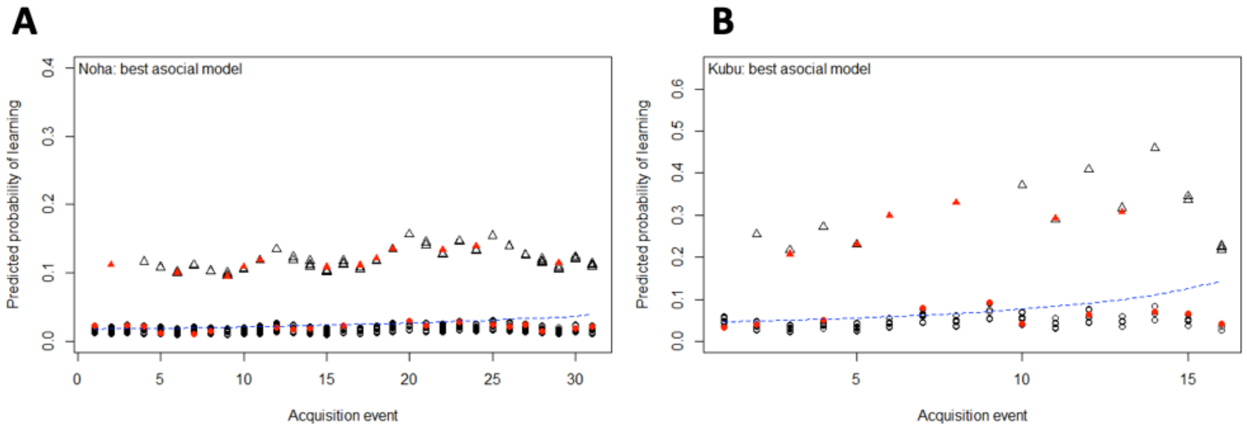

Supplementary Fig. 2. Plot representing the predicted probability of learning in function of the number of acquisition event for A) the best asocial for NH group and for B) the best asocial model for KB group. Each point is an individual x option combination. Triangles represent individuals who have already learned one option. Individuals who learned the task are in red. The blue line represents the average probability of learning across all individuals. The better the model fit, the more red points are above the blue line. Note that triangles tend to be plotted at a high probability, this means that an individual who has learned one option is more likely to learn the other option regardless its observational experience.

Supplementary Table 2. Model averaging testing for option-specific social learning. Variables with >50% support are shaded.

	Model-averaged estimate	Unconditional SE*	Back-transformed effect	Total Akaike weight
Social transmission per observation relative to baseline asocial learning rate				
Option specific (s_{OS})	0.237	0.096		0.951
Cross option (s_{CO})	0.019	0.017		0.337
**ILV effects on asocial learning**				
Other option solved	3.424	0.403	30.692	1.00
Option (pull-lift)	-0.585	0.442	0.557	0.569
Rank (lowest-highest)	0.032	0.237	1.033	0.243
Age (adult-nonadult)	0.034	0.065	1.035	0.232
Sex (female-male)	0.256	0.189	1.292	0.41
**ILV effects on social learning**				
Rank (lowest-highest)	-0.622	1.508	0.537	0.384
Age (adult-nonadult)	-0.152	241.835$	0.859	0.267
Sex (female-male)	0.764	72.288$	2.147	0.571

*Unconditional standard errors (UCSE) are approximate, see 'Validation of method for detecting option-specific social transmission' above. $The UCSE for age and sex effects on social learning are unrealistically high and are being skewed by a model or models with low weight. Consequently, in the main text we use 95% confidence intervals derived using the profile likelihood method to provide a plausible range for *s* parameters and ILV effects with support >50% (see Table 2, Main Text).

Dynamic observation networks and task exposure

It has been noted that there is concern with using dynamic observation networks where the target behaviour is performed in a specific location or locations- like here where the behaviour is directed to the foraging tasks provided [S5, S6]. A recorded observation for an individual *i* might simply indicate that *i* was in the area appropriate for performing/learning the behaviour, and consequently have been more likely to learn the behaviour in the near future. This might result in a spurious social transmission effect in an NBDA [S6]. A previous study [S5] addressed this by including a variable giving each individual's exposure to the task. However, in our case this is not necessary. Since the alternative options, 'lift' and 'pull', are performed in the same location, being recorded as an observer of 'lift' indicates that the observer was in the appropriate area for performing both 'lift' *and* 'pull', and likewise for observers of 'pull'. Therefore, we could only expect a spurious option-general effect. The fact that we find evidence of an option-specific effect rules out the possibility that it is a spurious effect of this kind.

Testing whether the learning generalization effect operated on asocial or social learning

The model described in Supplementary Note 1 assumes that the effect of solving one option on the rate of solving using a second option operates through asocial learning. The model was constructed in this way to reflect the hypotheses being tested- i.e. is the learned behaviour insulated against asocial modification? Strong support is found for this effect indicating that individuals would rapidly solve using the second option after solving using the first. However, it is premature to conclude that this effect operates by increasing the speed

at which the second option is learned by asocial learning, since we have not considered the possibility that the rate of social learning is increased instead or as well as that of asocial learning. Consequently, we re-fitted the best model, replacing the generalization effect on asocial learning ($+\beta_z z_{l,i}(t)$) with an equivalent effect on social learning ($+\gamma_z z_{l,i}(t)$). We found that AICc was increased by 6.63 units, indicating that the observed effect is much better explained by an increase in asocial learning rate than an increase in social learning rate. We also considered a model with effects on both social and asocial learning (AICc increased by 1.79) and with the same effect on both asocial and social learning ($\gamma_z = \beta_z$, AICc increased by 2.17) indicating that an effect on asocial learning alone is sufficient to explain the observed statistical pattern.

Overall these results indicate that the rate at which a second option, B, was learned, once an individual had learned a first option, A, was increased regardless of the individual's observational experience of option B. This suggests that learning a first option tended to be rapidly followed by asocial learning of a second option.

Supplementary Note 3: Transmission pathways analysis

Model description

We extended the model described in Supplementary Note 1 above to test for biases in the transmission pathways (Supplementary Table 2). Since there was strong support for OS social learning only (Supplementary Fig. 1), we simplified the model by dropping the CO effect. The biases we tested for were as follows:

a) Rank biases. Does transmission rate from higher to lower ranked vervet monkeys differ from that from lower to higher ranks?
b) Sex biases. Does the rate of transmission differ between male and female transmitters?
c) Age biases. Does the rate of transmission differ from adults to adults, adults to non-adults, non-adults to adults and non-adults to non-adults?
d) Kin biases. Does the rate of transmission differ between kin and non-kin, and between different classes of kin (mother to offspring, offspring to mother, between siblings)?

In a-b) the pathways are divided into only two mutually exclusive pathways: we first describe the procedure to test for rank biases, but the procedure can be generalized to b.

We obtained a binary network H_{ij}, taking the value 1 when *j* is a higher rank than *i* and 0 otherwise, thus representing the transmission pathway from higher to lower ranked vervet monkeys. The network $L_{ij} = 1 - h_{ij}$ therefore represented the pathway from lower to higher ranked vervet monkeys. This allowed us to extend the model by replacing the term:

$$s_{OS} \sum_{j} o_{ijl}(t)$$

With

$$s_{HL} \sum_{j} H_{ij} o_{ijl}(t) + s_{LH} \sum_{j} L_{ij} o_{ijl}(t)$$

Supplementary Equation 2.

Such that s_{HL} estimates the social transmission effect per observation of higher ranked vervet monkeys, whereas s_{LH} estimates the social transmission effect per observation of lower ranked vervet monkeys. We conducted multi-model inference as described above to quantify the support for rank bias ($s_{HL} \neq s_{LH}$) *versus* no rank bias ($s_{HL} = s_{LH}$), and also tested for transmission along each pathway only ($s_{HL} = 0$ and $s_{LH} = 0$).

When testing for an age bias we broke the observation network down into four mutually exclusive pathways: adult to adult; adult to non-adult; non-adult to adult; non-adult to non-adult, with the associated parameters: $s_{AA}, s_{AN}, s_{NA}, s_{NN}$. We then considered five different hypotheses, all pathways different ($s_{AA} \neq s_{AN} \neq s_{NA} \neq s_{NN}$), preferentially learn from older ($s_{AN} > s_{AA} = s_{NA} = s_{NN}$), only transmission from adults to non-adults ($s_{AA} = s_{NA} = s_{NN} = 0$), different transmission from older, younger and same age individuals ($s_{AA} = s_{NN} \neq s_{NA} \neq s_{AN}$), and no bias ($s_{AA} = s_{AN} = s_{NA} = s_{NN}$).

When testing for a kin bias we broke the observation network down into four mutually exclusive pathways: mother to offspring; offspring to mother; sibling to sibling; non-kin, with the associated parameters: s_{MO}, s_{OM}, s_S, s_N. We then considered three different hypotheses, all pathways different ($s_{MO} \neq s_{OM} \neq s_S \neq s_N$), kin different to non-kin ($s_{MO} = s_{OM} = s_S \neq s_N$) and no kin bias ($s_{MO} = s_{OM} = s_S = s_N$).

Additional results

Supplementary Table 3. Support for different pathways of transmission in an NBDA testing for sex, kin and age biases

**Transmission pathways**	**Total Akaike weight (%)**
**Sex biases**	
No bias	75.6
Different rates	24.4
**Kin biases**	
No bias	76.2
Kin different to non-kin	21.9
All pathways different (mother to offspring, offspring to mother, sibling to sibling, non-kin)	1.9
**Age biases**	
No bias	52.8
Older versus (younger or same age)	21.0
Transmission only from older	16.7
Older versus same age versus younger	6.7
All pathways different (adult to non-adult, non-adult to adult, adult to adult, non-adult to non-adult)	2.9

The following results are those of the same analyses as above but ran on pooling 'return and lift' (srtli) with 'lift' and 'return and pull' (srtpu) with 'pull' successes instead of 'lift' (sli) and 'pull' (spu)

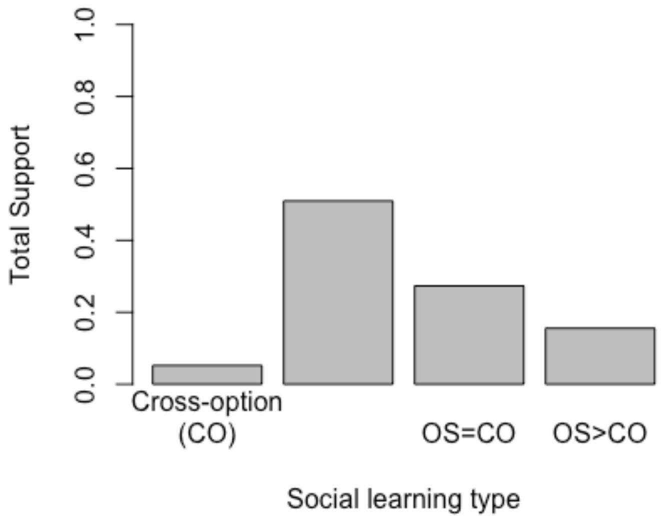

Supplementary Figure 3. Support for different combinations of option-specific (OS) and cross-option (CO) social learning. OS social learning received the most support, followed by models with separate *s* parameters for OS and CO social learning: in these cases, s_{OS} was estimated to be greater than s_{CO}. Overall these results indicate evidence that there was an OS social learning effect.

Supplementary Table 4. Model averaging testing for option-specific social learning. Variables with >50% support are shaded.

	Model-averaged estimate	Unconditional SE*	Back-transformed effect	Total Akaike weight
Social transmission per observation relative to baseline asocial learning rate				
Option specific (s_{OS})	0.084	0.010	NA	0.938
Cross option (s_{CO})	0.016	0.002	NA	0.481
**ILV effects on asocial learning**				
Other option solved	3.701	0.373	40.49	1.00
Option (pull-lift)	-0.724	0.343	0.485	0.739
Rank (lowest-highest)	-0.116	0.260	0.890	0.268
Age (adult-nonadult)	0.157	0.127	1.170	0.326
Sex (female-male)	0.003	0.074	1.003	0.246
**ILV effects on social learning**				
Rank (lowest-highest)	-1.483	4.093	0.227	0.567
Age (adult-nonadult)	-0.226	33.676$	0.798	0.314
Sex (female-male)	1.094	0.770	2.986	0.752

*Unconditional standard errors (UCSE) are approximate, see 'Validation of method for detecting option-specific social transmission' above. $The UCSE for age effect on social learning is unrealistically high and is being skewed by a model or models with low weight.

Supplementary Table 5. Support for different pathways of transmission in an NBDA testing for sex, kin, age and rank biases

Transmission pathways	Total Akaike weight (%)
**Sex biases**	
No bias	71.4
Different rates	28.6
**Kin biases**	
No bias	67.1
Kin different to non-kin	27.9
All pathways different (mother to offspring, offspring to mother, sibling to sibling, non-kin)	5.0
**Age biases**	
No bias	47.9
Older versus (younger or same age)	31.1
Transmission only from older	7.5
Older versus same age versus younger	10.4
All pathways different (mother to offspring, offspring to mother, sibling to sibling, non-kin)	3.1
**Rank biases**	
Higher to lower ranks only	54.1
Higher to lower > lower to higher	16.4
No bias	26.8
Lower to higher ranks only	2.7

Supplementary References

S1. R Core Team 2018. R: A language and environment for statistical computing. R Foundation for Statistical Computing. 3.5.2 ed. Vienna, Austria.

S2. Hoppitt, W., Photopoulou, T., Hasenjager, M., and Leadbeater, E. (2019). NBDA: A package for implementing Network-Based Diffusion Analysis. 0.7.10 ed.

S3. Franz, M., and Nunn, C.L. (2009). Network-based diffusion analysis: a new method for detecting social learning. Proc. R. Soc. B. 276, 1829-1836.

S4. Atton, N., Hoppitt, W., Webster, M. M., Galef, B. G., and Laland, K. N. (2012) 'Information flow through threespine stickleback networks without social transmission', Proc. Biol. Sci. *279(1745)*, 4272-4278.

S5. Hobaiter, C., Poisot, T., Zuberbühler, K., Hoppitt, W., and Gruber, T. (2014). Social network analysis shows direct evidence for social transmission of tool use in wild chimpanzees. PLos One. 12(9), e1001960.

S6. Hoppitt, W. (2017). The conceptual foundations of network-based diffusion analysis: choosing networks and interpreting results. Phil. Trans. R. Soc. B. *372*, 20160418.

S7. Kendal, R. L., Kendal, J. R., Hoppitt, W., and Laland, K. N. (2009) 'Identifying social learning in animal populations: a new 'option-bias' method'. *PLoS One,* 4(8), e6541.

S8. Barrett, B.J., McElreath, R.L. & Perry, S.E. Pay-off-biased social learning underlies the diffusion of novel extractive foraging traditions in a wild primate. *Proc. Biol. Sci*. **284**, 20170358 (2017)

S9. Hoppitt, W.J.E., Boogert, N.J., and Laland, K.N. (2010). Detecting social transmission in networks. J. Theor. Biol. 263(4), 544-555.

S10. Hoppitt, W., and Laland, K.N. (2013). Social learning: an introduction to mechanisms, methods, and models. Princeton University Press.

S11. de Vries, H. (1998). Finding a dominance order most consistent with a linear hierarchy: a new procedure and review. Anim. Behav. 55, 827-843.

S12. Burnham, K. P., and Anderson, D. R. (2002). Model Selection and Multimodel Inference: A Practical Information-Theoretic Approach. 2 edn. New York: Springer.

S13. Allen, J., Weinrich, M., Hoppitt, W., and Rendell, L. (2013). Network-based diffusion analysis reveals cultural transmission of lobtail feeding in humpback whales. Science. 340, 485-488.

S14. Farine, D.R., Spencer, K.A., and Boogert, N.J. (2015). Early-life stress triggers juvenile zebra finches to switch social learning strategies. Curr. Biol. *25*, 1-5.
