# Supplementary material for: Wild primates copy higher-ranked individuals in a social transmission experiment
Source: Nat Commun. 2020 Jan 23;11:459. doi: 10.1038/s41467-019-14209-8 (PMC6978360; doi:10.1038/s41467-019-14209-8)
Supplement: Supplementary file 4 — Description of Additional Supplementary Files [file 41467_2019_14209_MOESM4_ESM.pdf]

Description of Additional Supplementary Files

File Name: Supplementary Data 1
Description: The KUBU raw data file used for NBDA.

File Name: Supplementary Data 2
Description: The NOHA raw data file used for NBDA.

File Name: Supplementary Data 3
Description: The solving order matrix for KUBU.

File Name: Supplementary Data 4
Description: The solving order matrix for NOHA.

File Name: Supplementary Data 5
Description: The individual level variables for KUBU group.

File Name: Supplementary Data 6
Description: The individual level variables for NOHA group.

File Name: Supplementary Data 7
Description: The matrix displaying mother-offspring relationships used for NBDA in KUBU.

File Name: Supplementary Data 8
Description: The matrix displaying mother-offspring relationships used for NBDA in NOHA.

File Name: Supplementary Data 9
Description: The KUBU rank matrix (high rank/low rank).

File Name: Supplementary Data 10
Description: The NOHA rank matrix (high rank/low rank).

File Name: Supplementary Data 11
Description: The matrix displaying siblings relationships in KUBU.

File Name: Supplementary Data 12
Description: zthe matrix displaying siblings relationships in NOHA.

File Name: Supplementary Data 13
Description: The data file concerning individuals who attempted an succeeded to open the boxes which is used to run Supplementary Software 4.

File Name: Supplementary Data 14
Description: The data file concerning individuals who succeeded to open the boxes which is used to run Supplementary Software 4.

File Name: Supplementary Data 15
Description: The data file concerning observation in each dyad which is used to run Supplementary Software 5.

File Name: Supplementary Software 1
Description: The OADA script used to test for social transmission.

File Name: Supplementary Software 2
Description: The OADA script used to test for the different transmission pathways.

File Name: Supplementary Software 3
Description: The script used to run the simulations to test whether the 'lift' option was entirely due to an asocial bias.

File Name: Supplementary Software 4
Description: The script used to run the GLMs to test the effect of rank on the success rate.

File Name: Supplementary Software 5
Description: The script used to run the GLMMs to test the effect of rank on the observation rate.
