# Supplementary material for: Wild primates copy higher-ranked individuals in a social transmission experiment
Source: Nat Commun. 2020 Jan 23;11:459. doi: 10.1038/s41467-019-14209-8 (PMC6978360; doi:10.1038/s41467-019-14209-8)
Supplement: Supplementary file 20 — Supplementary Software [file 41467_2019_14209_MOESM20_ESM.zip › Supplementary Software/Instructions.rtf]

The attached script files 

Supplementary Software 1
Supplementary Software 2

are not software packages, but rather the code used to run the analyses described in the paper using the pre-existing and freely available NBDA package https://github.com/whoppitt/NBDA in the R statistical environment. Please see the citations in the main text for full references to these resources.

The following sections provide details to how to run the analysis as per the requirements of Nature publishing:

1. System requirements

Any machine with the R statistical environment installed, see https://www.r-project.org

2. Installation guide

Running the code requires prior installation of the R statistical environment, see  https://www.r-project.org
and the NBDA package, available at https://github.com/whoppitt/NBDA

To install the NBDA package install the package "devtools" in the usual way for R packages

Next load it up as follows:
library(devtools)

Then download and install my NBDA package from GitHub:
devtools::install_github("whoppitt/NBDA")

And load it as follows
library(NBDA)

3. Demo

The provided code is not a software package but a statistical analysis, so the code itself acts as a demo. This section of the readme is only included as per the requirements of Nature. Instructions to run on data, expected output and expected run time are covered in the following section.

4. Instructions for use

a) Save the provided script file and accompanying data files to the same directory on your computer.
b) Open R.
c) Open either script file Supplementary Software 1 or Supplementary Software 2 in R.
d) Change the working directory to the folder in which you have saved your files
e)  Execute each line of code- but please pay close attention to the comments within the code

Expected output: matching that presented in the comments in the code and in the supplementary material of the paper.
Expected runtime on a standard desktop PC: approximately 1 hour.
